# Transcriptome in paraffin samples for the diagnosis and prognosis of adrenocortical carcinoma

**DOI:** 10.1530/EJE-21-1228

**Published:** 2022-03-10

**Authors:** Anne Jouinot, Juliane Lippert, Mathilde Sibony, Florian Violon, Lindsay Jeanpierre, Daniel De Murat, Roberta Armignacco, Amandine Septier, Karine Perlemoine, Franck Letourneur, Brigitte Izac, Bruno Ragazzon, Karen Leroy, Eric Pasmant, Marie-Odile North, Sébastien Gaujoux, Bertrand Dousset, Lionel Groussin, Rossella Libe, Benoit Terris, Martin Fassnacht, Cristina L Ronchi, Jérôme Bertherat, Guillaume Assie

**Affiliations:** 1 Université de Paris, Institut Cochin, INSERM U-1016, CNRS UMR-8104, Paris, France; 2 Endocrinology, AP-HP Hôpital Cochin, Paris, France; 3 Institut Curie, INSERM U900, MINES ParisTech, PSL-Research University, CBIO-Centre for Computational Biology, Paris, France; 4 Division of Endocrinology and Diabetes, Department of Internal Medicine I, University Hospital, University of Wuerzburg, Wuerzburg, Germany; 5 Pathology, AP-HP Hôpital Cochin, Paris, France; 6 Genetics and Molecular Biology, AP-HP Hôpital Cochin, Paris, France; 7 Digestive and Endocrine Surgery, AP-HP Hôpital Cochin, Paris, France; 8 Institute of Metabolism and System Research, University of Birmingham, Birmingham, UK; 9 Centre for Endocrinology, Diabetes and Metabolism, Birmingham Health Partners, Birmingham, UK

## Abstract

**Design:**

Molecular classification is important for the diagnosis and prognosis of adrenocortical tumors (ACT). Transcriptome profiles separate adrenocortical adenomas ‘C2’ from carcinomas, and identify two groups of carcinomas ‘C1A’ and ‘C1B’, of poor and better prognosis respectively. However, many ACT cannot be profiled because of improper or absent freezing procedures, a mandatory requirement so far. The main aim was to determine transcriptome profiles on formalin-fixed paraffin-embedded (FFPE) samples, using the new 3’-end RNA-sequencing technology. A secondary aim was to demonstrate the ability of this technique to explore large FFPE archives, by focusing on the rare oncocytic ACT variants.

**Methods:**

We included 131 ACT: a training cohort from Cochin hospital and an independent validation cohort from Wuerzburg hospital. The 3’ transcriptome was generated from FFPE samples using QuantSeq (Lexogen, Vienna, Austria) and NextSeq500 (Illumina, San Diego, CA, USA).

**Results:**

In the training cohort, unsupervised clustering identified three groups: ‘C1A’ aggressive carcinomas (*n* = 28, 29%), ‘C1B’ more indolent carcinomas (*n* = 28, 29%), and ‘C2’ adenomas (*n* = 39, 41%). The prognostic value of FFPE transcriptome was confirmed in the validation cohort (5-year OS: 26% in ‘C1A’ (*n* = 26) and 100% in ‘C1B’ (*n* = 10), *P*  = 0.003). FFPE transcriptome was an independent prognostic factor in a multivariable model including tumor stage and Ki-67 (OS HR: 7.5, *P*  = 0.01). Oncocytic ACT (*n* = 19) did not form any specific cluster. Oncocytic carcinomas (*n* = 6) and oncocytic ACT of uncertain malignant potential (*n* = 4) were all in ‘C1B’.

**Conclusions:**

The 3’ RNA-sequencing represents a convenient solution for determining ACT molecular class from FFPE samples. This technique should facilitate routine use and large retrospective studies.

## Introduction

Adrenocortical carcinoma (ACC) is a rare endocrine cancer (1–2 per million per year) with an overall poor prognosis (1). Diagnosis is critical for patient management, but difficult owing to the rarity of the disease. Indeed, ACC is rare among adrenocortical tumors (ACT), the majority being adrenocortical adenomas (ACA). A pathology malignancy score called the Weiss score is most commonly used for diagnosis (2, 3). However, tumors with intermediate scores and rare histologic variants cannot be easily classified. In particular, determining the malignancy of oncocytic variants, composed of large cells with eosinophilic granulations, is challenging (4, 5).

The 5-year overall survival of ACC is generally below 40%, but heterogeneous (6). Prognostic stratification is important to guide treatment decision, especially in the adjuvant setting (7). The main prognostic factors used in clinical practice are tumor stage (8) and Ki-67 proliferation index (9). Other prognostic factors commonly include cortisol secretion (10) and age (11).

Previous genomic studies have demonstrated a convergence of genomic alterations into distinct molecular subtypes (12, 13). Transcriptome profiles represent the cornerstone of molecular classification. Transcriptome profiles separate adrenocortical adenomas (C2) from carcinomas and identify two groups of ACC strongly associated with outcome: the ‘C1A’ subgroup, with an overexpression of proliferative genes and a poor prognosis, and the ‘C1B’ subgroup, with an immune signature and a better prognosis (12, 13, 14, 15). Specific mRNA markers, such as the differential gene expression of *BUB1B-PINK1*, have then been deduced from transcriptome signatures. These targeted markers improve diagnosis and prognostic assessment of ACC (16, 17, 18). However, transcriptome profiles and their targeted surrogate markers are up to now limited to high-quality tumor RNA, requiring high-quality frozen tissue samples. Obtaining this type of material is demanding in routine practice, and often not achieved for ACC patients.

The recent 3’ RNA-sequencing technology captures and sequences short fragments at the 3’ end of poly-adenylated RNA, which are less prone to RNA degradation. Unlike gene expression microarrays or standard full-length RNA-sequencing, this technique requires only a low amount and low-quality RNA, compatible with formalin-fixed paraffin-embedded (FFPE) samples (19).

In this study, we performed 3’ RNA-sequencing on FFPE samples of adrenocortical tumors. We evaluated the diagnostic and prognostic value of FFPE transcriptome classification, investigated the transcriptome classification of oncocytic variants, and assessed intra-tumor heterogeneity.

## Subjects and methods

### Patients

A training cohort of 95 ACT patients from Cochin Hospital (Paris, France) was used to explore FFPE transcriptome profiles of classical ACC, ACA and oncocytic ACT. All consecutive primary tumor specimens of classical ACC and oncocytic ACT collected between 2008 and 2018 were included. A control group of classical ACA was randomly selected over the same period. A fragment of tumor tissue was frozen in liquid nitrogen immediately after surgery and the remaining tumor was fixed in formalin and embedded in paraffin. The diagnosis of malignancy was established by an experienced endocrine pathologist (MS and FV) using the Weiss score (2, 3) (ACC if ≥3 malignancy criteria) or the Lin–Weiss–Bisceglia score (4) for oncocytic tumors (ACC if ≥1 major criteria, ACT of undetermined malignant potential if ≥1 minor criteria). For eight patients, two different regions of the primary tumor were sampled to assess the intratumor heterogeneity of ACT transcriptome. For 5/8 tumors, distinct nuclear and cytologic features were observed between the two regions.

A validation cohort of 36 ACC patients from Wuerzburg University Hospital (Wuerzburg, Germany) was then used to test the prognostic value of FFPE transcriptome profiles.

Clinical information was obtained from the European Network for the Study of Adrenal Tumors (ENSAT) registry (20) (Supplementary Table 1, see section on supplementary materials given at the end of this article).

Written consent has been obtained from each patient or subject after a full explanation of the purpose and nature of all procedures used. The study was conducted in accordance with the Declaration of Helsinki and approved by the Comité de protection des personnes Ile de France 1 (application no. 13311) and the Ethics Committee of the University of Wuerzburg (registration no. 88/11).

### Targeted PCR marker on frozen samples

The differential gene expression of *BUB1B*-*PINK1*, recapitulating the ‘C1A’ and ‘C1B’ transcriptome difference, was used to predict ‘poor-outcome’ and ‘better-outcome’ ACC, as previously published (15, 16).

### 3’ RNA-sequencing on FFPE samples

FFPE material was selected by an experienced endocrine pathologist. RNA extraction was performed from 10 × 6 µm FFPE tissue sections using the Maxwell 16 LEV RNA FFPE kit and a Maxwell 16 Instrument (Promega). RNA was quantified using a Qubit fluorometer (Thermo Fisher Scientific) and analyzed by electrophoresis on a Bioanalyser 2100 (Agilent Technologies). As expected, FFPE-extracted RNA showed degraded profiles, with RNA Integrity Number <3. No selection based on RNA quality was carried out.

Sequencing libraries were prepared at the Genomics Platform of the Cochin Institute, following the QuantSeq 3’ mRNA-Seq protocol (Lexogen, Vienna, Austria), starting from 100 to 500 ng RNA. Single read sequencing (1 ×75bp) was performed on a NextSeq 550 platform (Illumina, San Diego, CA, USA).

### Transcriptome analyses

FASTQ sequences were aligned on GRCh38 human reference genome with STAR (v.2.7.3a) (21). Read counts (Supplementary Table 2) were normalized with DESeq2 (v.1.28.1) (22). Genes were filtered by s.d. to select the 2000 most variable genes for subsequent analyses (classification, principal component analysis, and transcriptome prediction).

Unsupervised classification of ACT transcriptome profiles was performed in the 95 patients of the Cochin cohort. Among tumors with two distinct regions, only the most histologically aggressive region was included in unsupervised classification. Groups with similar gene expression profiles were identified using non-negative matrix factorization (NMF) consensus clustering with 200 iterations, implemented in the NMF R package (v0.22.0) (23). The best rank of factorization (k = 3) was chosen from both cophenetic and average silhouette width score profiles. Adrenal differentiation, proliferation and immune signatures (Supplementary Table 3) were previously proposed to characterize transcriptome subgroups in the ACC TCGA study (13). The overall level of activation of these signatures was calculated in each sample using a single sample GSEA implemented in GSVA R package (v1.36.2) (24). Differentially expressed genes between NMF groups were identified using the Kruskal–Wallis test, with Benjamini-Hochberg correction for multiple testing. Gene-set enrichment analyses were performed with the GSEA pre-ranked method (v4.0.3) (25), using the ‘Hallmarks’ annotation v7.1 (MSigDB).

Principal component analysis (PCA) was performed to visualize individuals in the two-dimension views that capture the greatest amount of variability, using factoextra (v1.0.6) and FactoMineR (v2.3) R packages (26). The 95 unique ACT from Cochin were used to compute PCA. ACT with two distinct tumor regions and ACC from the validation cohort was then projected as supplementary individuals on the same representation.

Finally, a transcriptome-based model was developed to classify any new ACC sample into C1A/C1B prognostic groups. Read counts were normalized on the sequencing depth only using the formula:


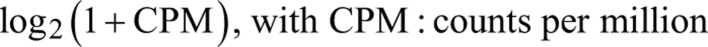


With this normalization, additional samples can be processed independently from the original cohort. A ridge penalized regression with ten-fold cross-validation was applied to predict the C1A/C1B status on the training (Cochin) cohort, using the glmnet R package (v4.0-2) (27). This model was then applied to the validation (Wuerzburg) cohort.

### Statistical analysis

Comparisons between groups were performed with the Wilcoxon and Kruskal–Wallis tests for quantitative variables, and with Fisher’s test for qualitative variables.

Disease-free survival (DFS) was analyzed in patients with ENSAT stage I–III ACC and was defined as the time from primary tumor resection until recurrence or last follow-up. Overall survival (OS) was analyzed in stage I–IV ACC, and defined as the time from pathological diagnosis until death or last follow-up. Survival curves were obtained with Kaplan–Meier estimates and compared with a log-rank test. Cox proportional hazards regression was used to identify variables associated with DFS and OS. The proportional hazards assumption was checked for each model using graphical methods based on Kaplan–Meier curves and the scaled Schoenfeld residuals. Significant variables were combined into multivariable models.

All *P* -values were two-sided, and the level of significance was set at *P*  < 0.05. Calculations were performed using R statistical software (v4.1.1, R Stats, survival and survcomp packages).

## Results

### Patient characteristics

A training cohort of 95 ACT patients from Cochin hospital (43 classical ACC, 33 classical ACA and 19 oncocytic ACT), and an independent validation cohort of 36 ACC patients from Wuerzburg hospital were included (Table 1 and Supplementary Table 1). Median follow-up were 40.3 months (95% CI: 2.1–136.1) in the training cohort and 30.5 months (95 CI: 4.0–225.9) in the validation cohort.

**Table 1:** Patients’ characteristics. Values are expressed in *n* (%) for qualitative variables and in median (range) for quantitative variables. *P*-values *<* 0.05 are indicated in bold.

	Training cohort		Validation cohort	*P*-value^†^
	ACA	ACC	ACC	
*n*	42	49 + 4*	36	
Age (years)	51 (25–85)	47 (21–73)	49 (20–81)	0.34
Sex				0.33
Female	33 (79%)	41 (77%)	24 (67%)	
Male	9 (21%)	12 (23%)	12 (33%)	
Cortisol secretion				0.46
Yes	32 (78%)	33 (62%)	13 (52%)	
No	9 (22%)	20 (38%)	12 (48%)	
Not available	1		11	
ENSAT stage				0.82
I	33 (82%)	8 (15%)	3 (8%)	
II	7 (18%)	26 (49%)	18 (50%)	
III		12 (23%)	10 (28%)	
IV		7 (13%)	5 (14%)	
Not available	2			
Subtype				**0.005**
Classical	33 (79%)	43 (81%)	36 (100%)	
Oncocytic	9 (21%)	10 (19%)	0 (0%)	
Resection				
R0	42 (100%)	46 (87%)	24 (67%)	0.08
R1		2 (4%)	3 (8%)	
R2		0 (0%)	2 (6%)	
RX		5 (9%)	7 (19%)	
Weiss score				0.17
0–1	26 (62%)			
2	13 (31%)			
3	3 (7%)	8 (15%)	1 (3%)	
4–6		17 (32%)	14 (47%)	
7–9		28 (53%)	15 (50%)	
Ki-67				**0.008**
<10%	29 (100%)	23 (49%)	6 (17%)	
10–19%	0 (0%)	7 (15%)	10 (28%)	
≥20%	0 (0%)	17 (36%)	20 (56%)	
Not available	13	6		
Relapse or metastases				<10**−4**
No	34 (100%)	33 (62%)	6 (17%)	
Yes	0 (0%)	20 (38%)	30 (83%)	
Not available	8			
Death				0.27
No	34 (100%)	36 (68%)	20 (56%)	
Yes	0 (0%)	17 (32%)	16 (44%)	
Not available	8			

*ACC (*n* = 49) and ACT of uncertain malignant potential (*n* = 4); **^†^***P*-values are provided for the comparison between ACC and ACT of uncertain malignant potential from the training cohort and ACC from the validation cohort.

### Impact of sample age on sequencing data

The median Q30 per sample was 88%, ranging from 84−91%. A median of 11 665 648 aligned reads per sample (range 3 255 802−22 861 798) were obtained. Sample age may impact both the amount (median number of aligned reads 12 780 317 in the 10 oldest samples vs 16 853 879 in the 10 most recent samples, *P*  = 0.001) and quality of reads (median Q30 87% in the 10 oldest samples vs 90% in the 10 most recent samples, *P*  < 10−4). In spite of slightly lower quality, transcriptome profiles were not impacted, since no association was observed between transcriptome classification and sample age (data not shown). Moreover, no technical failures were observed, even for samples collected 10 years ago.

### FFPE transcriptome classification of ACT

**Figure 1:**
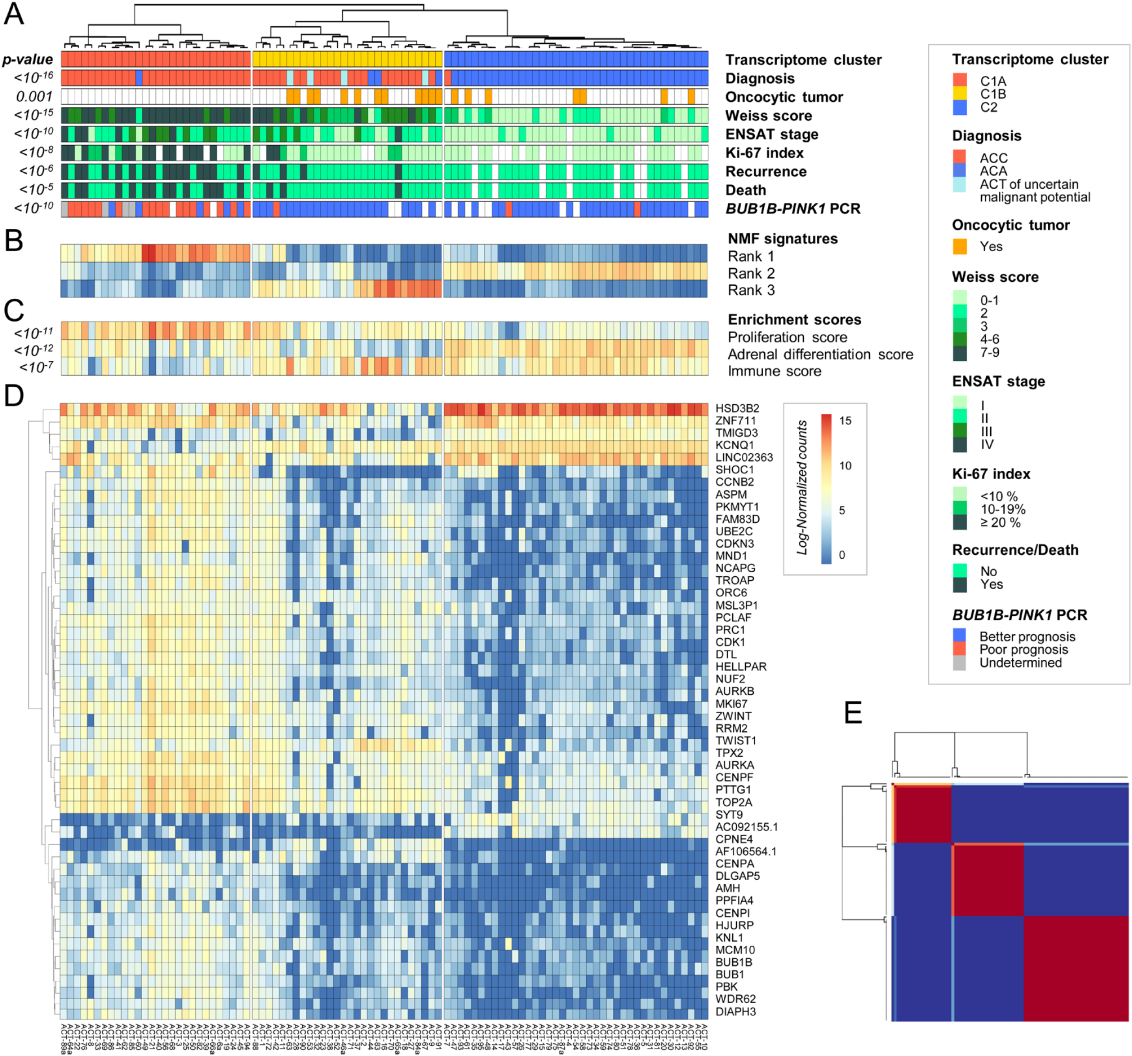
FFPE transcriptome classification of ACT in the training cohort. (A) Unsupervised classification of ACT based on the 2000 most variable genes identifies three main groups, corresponding to ‘C1A’ aggressive carcinomas, ‘C1B’ indolent carcinomas and ‘C2’ adenomas. (B) Heatmap of the three non-negative matrix factorization (NMF) ranks used for generating the unsupervised classification. (C) Expression of steroid (adrenal differentiation), proliferation and immune signatures in each ACT. (D) Expression profiles of the top 50 most significantly different genes among the three transcriptome groups. (E) Consensus matrix representing the similarity between samples over iterative clustering algorithms. Rows and columns are patient samples. Consensus matrix values range from 0 (never clustered together) to 1 (always clustered together).

In the training cohort, unsupervised consensus clustering of ACT transcriptome identified three distinct groups, closely associated with clinical, pathological and molecular characteristics (Fig. 1): (i) a group of aggressive ACC, enriched in advanced, high-grade tumors, showing high rates of recurrence (16/28, 57%) and death (14/28, 50%). This group was classified as poor-outcome with the targeted PCR marker *BUB1B-PINK1*; (ii) a group of more indolent ACC, enriched in localized, low-grade tumors, showing low rates of recurrence (4/28, 14%) and death (3/28, 11%). This group was classified as better-outcome with the targeted PCR marker *BUB1B-PINK1*; (iii) a group of ACA.

In terms of gene expression profiles, these FFPE transcriptome groups correspond to the known ACT classes (12, 13, 15) (Fig. 1A, B, C, D, E and Supplementary Tables 4, 5), with (i) high expression of cell-cycle genes such as *TOP2A*, *MKI67* or *BUB1B* and enrichment in g2m_checkpoint and e2f_targets gene sets in the aggressive ACC group, previously referred to as the ‘C1A’ transcriptome class; (ii) an immune signature, with enrichment in allograft_rejection, interferon_gamma_response and il6_jak_stat3_signaling gene sets in the more indolent ACC group, previously referred to as the ‘C1B’ transcriptome class; (iii) high adrenal differentiation scores, and high expression of steroid enzymes in the ACA group, previously referred to as the ‘C2’ transcriptome class.

### FFPE transcriptome profiles of oncocytic ACT

In this cohort, 19/95 ACT were oncocytic tumors, including 6 ACC, 4 ACT of uncertain malignant potential and 9 ACA according to the Lin–Weiss–Bisceglia classification (4). In unsupervised transcriptome classification, these ACT did not segregate into any specific cluster of oncocytic tumors. Instead, oncocytic ACT scattered, evenly mixed with other ACT within C2 adenomas and C1B carcinomas (Fig. 1A), with no specific transcriptome signature (Supplementary Table 6). In particular, all oncocytic ACC and oncocytic ACT of uncertain malignant potential clustered with C1B carcinomas.

### FFPE transcriptome profiles in different tumor regions

**Figure 2:**
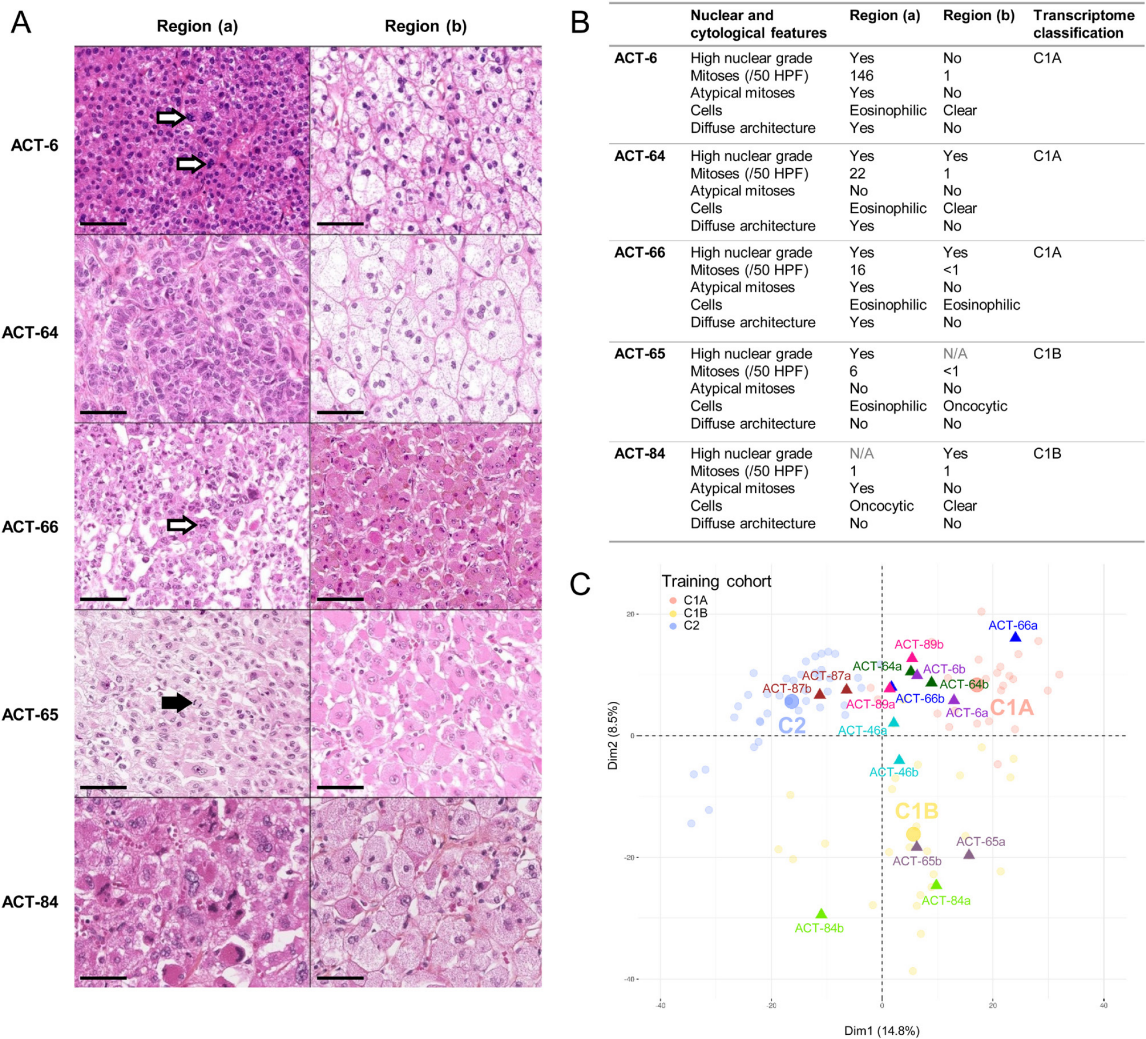
FFPE transcriptome classification in different tumor regions. (A) Hematoxylin/eosin/safran staining (40× magnification) of the two different tumor regions sampled for five patients in the training cohort, with one aggressive (region a) and one less aggressive (region b) region. The black bar represents 50 µm. Black arrows indicate mitosis. White arrows indicate atypical mitoses. (B) Description of the two different tumor regions sampled for the five patients with one aggressive (-a) and one less aggressive (-b) region. N/A: Fuhrman nuclear grade is not applicable for oncocytic cells. (C) Samples projection based on the two principle components (Dim1 and Dim2) of the PCA performed based on the 2000 most variable genes in the 95 unique patients of the training cohort. Samples from the training cohort are presented as faint circles colored by transcriptome class. Samples from two different tumor regions for eight patients are presented as full triangles colored by patient.

Transcriptome heterogeneity was explored in two different regions of the primary tumor for eight patients (Fig. 2A and B). For all patients, the two samples were concordant in terms of transcriptome classification, in spite of distinct nuclear and cytological features for 5/8 tumors (Fig. 2C). For these patients, tumor regions with mainly ‘benign’ features on pathology were properly classified as carcinomas (C1A or C1B), following the transcriptome class of their most aggressive counterpart (Fig. 2C).

### FFPE transcriptome prognostic value

**Figure 3:**
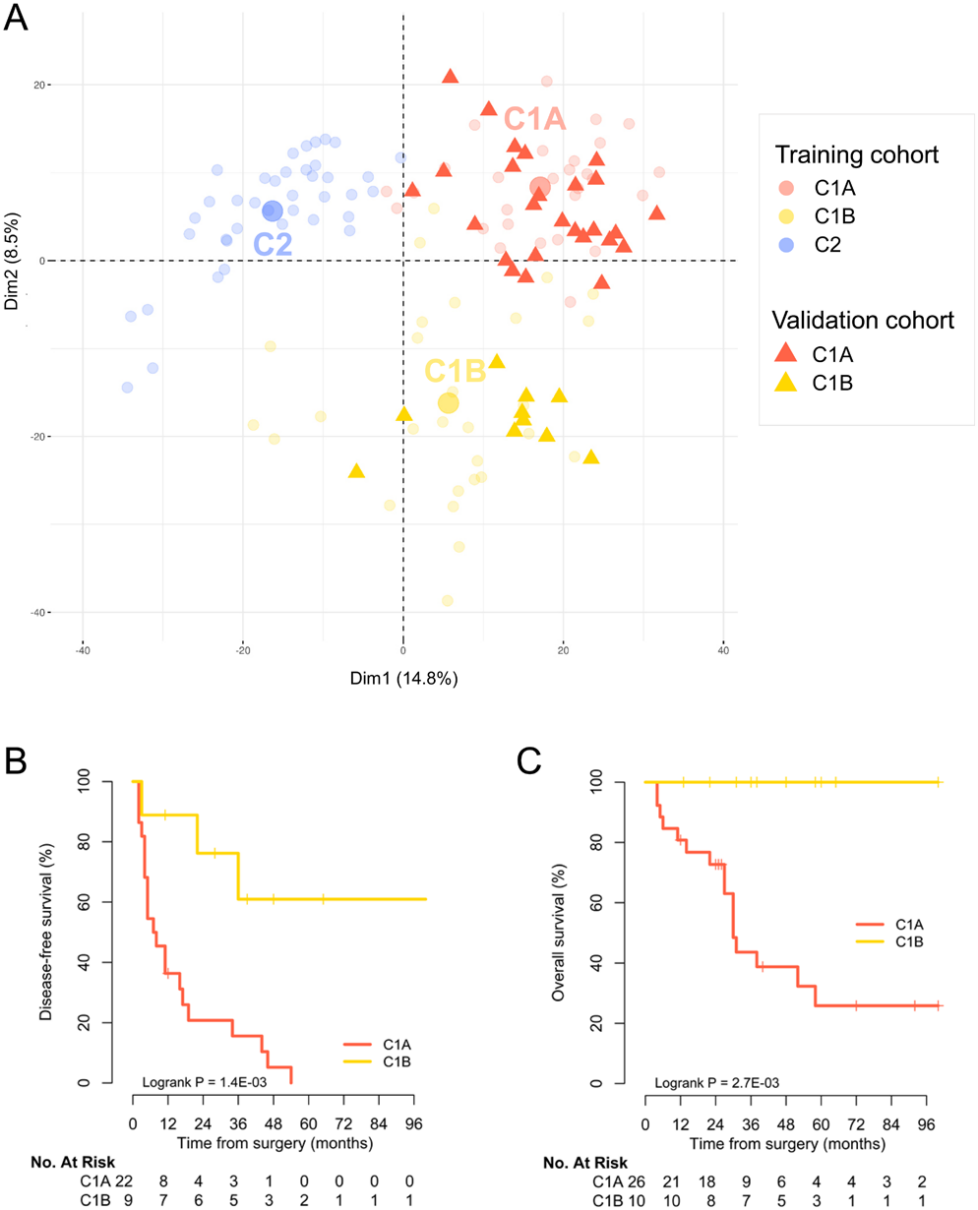
FFPE transcriptome classification and prognosis in the ACC validation cohort. (A) Samples projection based on the two principle components (Dim1 and Dim2) of the PCA performed based on the 2000 most variable genes in the 95 unique patients of the training cohort. Samples from the training cohort are presented as faint circles colored by transcriptome class. Samples from validation cohort are presented as full triangles colored by transcriptome class. (B) Disease-free survival according to the predicted transcriptome class in stage I–III ACC patients of the validation cohort. (C) Overall survival according to the predicted transcriptome class in stage I–IV ACC patients of the validation cohort.

C1A and C1B carcinomas showed a distinct outcome (Supplementary Fig. 1), as previously established (12, 13, 15, 16). FFPE transcriptome prognostic value through proper C1A/C1B classification was confirmed in an independent validation cohort. Transcriptome profiles of these independent samples were close to either C1A (*n* = 26) or C1B (*n* = 10) carcinomas from the training cohort (Fig. 3A). We next developed a predictive model of transcriptome class, using cross-validated ridge regression, for determining the prognosis of individual patients. This model was optimized to discriminate C1A from C1B carcinomas on the training cohort. On the validation cohort, predicted C1A carcinomas were associated with shorter DFS (median 7.5 vs 240 months, *P*  = 0.0014) and OS (median 30 months vs not reached, *P*  = 0.0027) compared to predicted C1B carcinomas (Fig. 3B and C). Of note, 5-year OS was 26% (95 CI 12 to 56%) in predicted C1A and 100% in predicted C1B carcinomas.

### Multivariate prognostic analyses

The prognostic value of FFPE transcriptome classification was then tested against known ACC prognostic factors in the whole ACC cohort.

C1A cluster was associated with cortisol-secreting tumors (*P*  = 0.001), high Ki-67 (*P*  < 10^−7^) and incomplete surgical resection (*P*  = 0.0002) (Supplementary Table 7).

In stage I–III ACC, ENSAT stage, Ki-67 index and molecular class were identified as independent prognostic factors of DFS (Table 2). In stage I–IV ACC, ENSAT stage and molecular class were the only independent prognostic factors of OS (Table 3).

**Table 2:** Disease-free survival analyses in stage I-III ACC patients. *P*-values *<* 0.05 are indicated in bold.

	Univariate analysis			Multivariable analysis		
	HR	95% CI	*P*-value	HR	95% CI	*P*-value
Age (per year increase)	1.02	1.00–1.04	0.11			
Sex (M vs F)	0.92	0.45–1.86	0.81			
Cortisol secretion (Yes vs no)	1.81	0.86–3.80	0.12			
ENSAT stage (III vs I–II)	3.31	1.72–6.38	**0.0003**	3.38	1.61–7.12	**0.001**
Resection status (R1–R2–RX vs R0)	4.35	2.12–8.93	<10**−4**	1.36	0.61–3.00	0.45
Ki-67 proliferation index						
10–19% vs <10%	2.85	0.92–8.88	0.07	1.76	0.52–5.96	0.36
≥20% vs <10%	7.63	3.08–18.93	<10**−4**	3.12	1.02–9.57	**0.046**
Molecular class (C1A vs C1B)	6.98	2.89–16.87	<10**−4**	3.39	1.09–10.55	**0.035**

**Table 3:** Overall survival analyses in stage I-IV ACC patients. *P*-values *<* 0.05 are indicated in bold.

	Univariate analysis			Multivariable analysis		
	HR	95% CI	*P*-value	HR	95% CI	*P*-value
Age (per year increase)	1.01	0.99–1.04	0.40			
Sex (M vs F)	0.51	0.21–1.23	0.13			
Cortisol secretion (yes vs no)	4.04	1.62–10.07	**0.003**	1.16	0.32–4.13	0.82
ENSAT stage						
III vs I–II	5.68	2.44–13.23	<10**−4**	8.19	2.41–27.81	**0.0007**
IV vs I–II	10.95	4.35–27.54	<10**−6**	32.34	7.03–148.86	<10**−5**
Resection status (R1–R2–RX vs R0)	4.88	2.39–9.98	<10**−4**	0.97	0.35–2.66	0.95
Ki-67 proliferation index						
10–19 % vs <10%	2.96	0.66–13.27	0.16	0.81	0.15–4.28	0.80
≥20% vs <10%	10.20	3.05–34.12	**0.0002**	1.79	0.35–9.00	0.48
Molecular class (C1A vs C1B)	10.15	3.09–33.35	**0.0001**	7.5	1.47–38.13	**0.01**

## Discussion

This study shows the feasibility of ACT transcriptome from FFPE samples. All samples were suitable for the technique, even those collected more than 10 years ago. We showed that diagnostic and prognostic transcriptome classification of ACT can be obtained from FFPE samples using 3’ RNA-sequencing. We were able to assess the transcriptome classification of oncocytic ACT and the intratumor heterogeneity of ACT transcriptome and to develop a new transcriptome-based model for ACC prognostication in clinical routine.

Oncocytic variants are representing approximatively 10% of ACT (28). The diagnosis of malignancy and the prognostic assessment for these tumors are particularly challenging. A specific malignancy score referred to as the Lin-Weiss-Bisceglia score has been specifically developed for oncocytic ACT (4, 29). When malignancy is confirmed, several studies suggest that oncocytic ACC is associated with better prognosis than conventional ACC (5, 29). However, diagnostic and prognostic criteria for oncocytic ACT have been established only in small clinical series, and these tumors have never been specifically explored in pangenomic studies. In our study, oncocytic ACT did not form any specific subgroup, suggesting that the oncocytic feature is not translating any different tumor biology. Instead, oncocytic ACT all clustered within C1B and C2 clusters. A good agreement with LinWeiss–Bisceglia score was observed, with the noticeable classification of all oncocytic ACT of uncertain malignant potential within C1B carcinomas. Of note, for non-oncocytic ACT, 6/7 ACT with Weiss score of 2 and 1/4 with Weiss score of 3 clustered in C2 adenomas. This confirms the uncertain malignant potential of non-oncocytic ACT with Weiss scores between 2 and 3.

Several studies demonstrated the importance of molecular classification for ACC prognostic stratification (13, 14, 15, 16, 18). However, transcriptome profiles and their targeted surrogates were so far limited to high-quality frozen tumor samples, precluding their use in routine practice. Moving to FFPE samples is therefore an important step. In the current study, we proposed a transcriptome-based model to classify any new ACC sample into C1A/C1B prognostic groups. The prognostic value of FFPE transcriptome classification could be validated on an independent ACC cohort. Finally, FFPE transcriptome classification was confirmed as an independent prognostic factor in multivariable models including ENSAT stage and tumor grade.

Intratumor heterogeneity is well established in ACC, both in terms of pathology (30, 31), and somatic mutations (32, 33), while DNA methylation and chromosomal alteration profiles seem less heterogeneous (33). This study provides the first evidence of transcriptome homogeneity in different tumor regions. Remarkably, for five patients presenting one aggressive and one less aggressive tumor region, with mainly ‘benign’ features on pathology, transcriptome classification was “malignant” for both components. This is in agreement with current pathological practice, which takes into account the most aggressive tumor part for the diagnosis. The impact of sampling regions on transcriptome classification seems limited. This finding is important for using transcriptome information in clinical decisions.

This study presents some limitations. First, the sample size is limited. Further studies on larger cohorts are needed to confirm our results, especially regarding the transcriptome of oncocytic tumors and intratumor heterogeneity. However, ACC are rare, and this study included 89 ACC samples from two distinct centers. In addition, the prognostic value of FFPE transcriptome could be validated on a cohort independent from the training cohort used to establish the transcriptome classification. Secondly, a gold standard is missing for malignancy diagnosis of ACT. Indeed the current pathological scoring systems (Weiss and Lin–Weiss–Bisceglia) were established by comparing recurrent (metastazing) to non-recurrent ACT in small series of patients (2, 3, 4, 29). However, whole transcriptome classification is unsupervised and respects the data structure reflecting tumor biology.

Starting from FFPE samples, we used a 3’-end RNA-sequencing transcriptome technology. This technology is robust, cheaper and simpler to run compared to full-length RNA-sequencing (19). Other gene expression techniques starting from FFPE have been reported in ACC (34, 35, 36). However, these techniques have not emerged yet as optimal for prognostic determination in ACC. The 3’ RNA-sequencing recently emerged as an opportunity to study transcriptome signatures from FFPE material, as reported for several tumor types (37, 38, 39). This technique was applied to samples from operated patients. Whether it would be applicable to smaller amounts of tissue material, such as those from core biopsies, remains to be established. In this study, we based our classification of individual samples on the global transcriptome signature, a robust feature even when some individual markers are not detected because of poor RNA quality. Other advantages of FFPE samples use include an easier transfer of samples to oncogenetic departments in routine practice, the ability to optimize the tumor region to be analyzed, and the inclusion of large archive collections.

In conclusion, reliable transcriptome classification for diagnosis and prognosis of ACT can be determined from standard FFPE samples using the recent 3’ RNA-sequencing technologies. This novel approach unraveled the molecular specificities of oncocytic variants. Transcriptome classification was robust in spite of intratumor heterogeneity. Finally, an accurate predictive model of transcriptome class was designed and validated for the classification of any new sample. This study provides researchers and clinicians with a convenient solution for the determination of ACT transcriptome from FFPE samples.

## Supplementary Material

Supplementary Data

Supplementary Table S1. Individual patient clinical data.

Supplementary Table S2. Individual transcriptome data.

Supplementary Table S4. Differentially expressed genes between transcriptome groups.

Supplementary Table S5. Gene set enrichment analyses between transcriptome groups using HALLMARK annotations.

Supplementary Table S6. Differentially expressed genes between oncocytic and classic ACT.

## Declaration of interest

Martin Fassnacht and Guillaume Assie are on the editorial board of EJE. They were not involved in the review or editorial process for this paper, on which they are listed as authors. The other authors have nothing to disclose.

## Funding

This work was supported by the Programme de Recherche Translationnelle en Cancérologie to the COMETE network (PRT-K COMETE-TACTIC and PRT-K COMETE-CARE), the CARPEM (CAncer Research for PErsonalized Medicine), the ITMO Cancer of AVIESAN (Alliance pour les Sciences de la Vie et de la Santé) on funds administrated by INSERM (A J received a PhD and post-doctoral grant), the Deutsche Krebshilfe http://dx.doi.org/10.13039/501100005972 (70112969 to C L R and M F) and Deutsche Forschungsgemeinschaft http://dx.doi.org/10.13039/501100001659 (no. 314061271 – CRC/TRR 205, .FA-466/4-2 and FA-466/8-1 to M F, and RO-5435/3-1 to C L R).

## Author contribution statement

J Bertherat and G Assie jointly supervised the work reported here.

## References

[1] Kerkhofs TMA Verhoeven RHA Van der Zwan JM Dieleman J Kerstens MN Links TP Van de Poll-Franse LV Haak HR. Adrenocortical carcinoma: a population-based study on incidence and survival in the Netherlands since 1993. European Journal of Cancer 2013 49 2579–2586. (10.1016/j.ejca.2013.02.034) 23561851

[2] Ayala-Ramirez M Jasim S Feng L Ejaz S Deniz F Busaidy N Waguespack SG Naing A Sircar K Wood CG***et al***. Adrenocortical carcinoma: clinical outcomes and prognosis of 330 patients at a tertiary care center. European Journal of Endocrinology 2013 169 891–899. (10.1530/EJE-13-0519) 24086089 PMC4441210

[3] Fassnacht M Assie G Baudin E Eisenhofer G de la Fouchardiere C Haak HR de Krijger R Porpiglia F Terzolo M Berruti A***et al***. Adrenocortical carcinomas and malignant phaeochromocytomas: ESMO-EURACAN Clinical Practice Guidelines for diagnosis, treatment and follow-up. Annals of Oncology 2020 31 1476–1490. (10.1016/j.annonc.2020.08.2099) 32861807

[4] Weiss LM Comparative histologic study of 43 metastasizing and nonmetastasizing adrenocortical tumors. American Journal of Surgical Pathology 1984 8 163–169. (10.1097/00000478-198403000-00001) 6703192

[5] Renaudin K Smati S Wargny M Al Ghuzlan A Aubert S Leteurtre E Patey M Sibony M Sturm N Tissier F***et al***. Clinicopathological description of 43 oncocytic adrenocortical tumors: importance of Ki-67 in histoprognostic evaluation. Modern Pathology 2018 31 1708–1716. (10.1038/s41379-018-0077-8) 29921900

[6] Bisceglia M Ludovico O Di Mattia A Ben-Dor D Sandbank J Pasquinelli G Lau SK Weiss LM. Adrenocortical oncocytic tumors: report of 10 cases and review of the literature. International Journal of Surgical Pathology 2004 12 231–243. (10.1177/106689690401200304) 15306935

[7] Fassnacht M Johanssen S Quinkler M Bucsky P Willenberg HS Beuschlein F Terzolo M Mueller HH Hahner S Allolio B***et al***. Limited prognostic value of the 2004 International Union against cancer staging classification for adrenocortical carcinoma: proposal for a revised TNM classification. Cancer 2009 115 243–250. (10.1002/cncr.24030) 19025987

[8] Beuschlein F Weigel J Saeger W Kroiss M Wild V Daffara F Libé R Ardito A Al Ghuzlan A Quinkler M***et al***. Major prognostic role of Ki67 in localized adrenocortical carcinoma after complete resection. Journal of Clinical Endocrinology and Metabolism 2015 100 841–849. (10.1210/jc.2014-3182) 25559399

[9] Weiss LM Medeiros LJ Vickery AL. Pathologic features of prognostic significance in adrenocortical carcinoma. American Journal of Surgical Pathology 1989 13 202–206. (10.1097/00000478-198903000-00004) 2919718

[10] Vanbrabant T Fassnacht M Assie G Dekkers OM. Influence of hormonal functional status on survival in adrenocortical carcinoma: systematic review and meta-analysis. European Journal of Endocrinology 2018 179 429–436. (10.1530/EJE-18-0450) 30325179

[11] Libé R Borget I Ronchi CL Zaggia B Kroiss M Kerkhofs T Bertherat J Volante M Quinkler M Chabre O***et al***. Prognostic factors in stage III–IV adrenocortical carcinomas (ACC): an European Network for the Study of Adrenal Tumor (ENSAT) study. Annals of Oncology 2015 26 2119–2125. (10.1093/annonc/mdv329) 26392430

[12] Assié G Letouzé E Fassnacht M Jouinot A Luscap W Barreau O Omeiri H Rodriguez S Perlemoine K René-Corail F***et al***. Integrated genomic characterization of adrenocortical carcinoma. Nature Genetics 2014 46 607–612. (10.1038/ng.2953) 24747642

[13] Zheng S Cherniack AD Dewal N Moffitt RA Danilova L Murray BA Lerario AM Else T Knijnenburg TA Ciriello G***et al***. Comprehensive pan-genomic characterization of adrenocortical carcinoma. Cancer Cell 2016 29 723–736. (10.1016/j.ccell.2016.04.002) 27165744 PMC4864952

[14] Giordano TJ Kuick R Else T Gauger PG Vinco M Bauersfeld J Sanders D Thomas DG Doherty G Hammer G. Molecular classification and prognostication of adrenocortical tumors by transcriptome profiling. Clinical Cancer Research 2009 15 668–676. (10.1158/1078-0432.CCR-08-1067) 19147773 PMC2629378

[15] de Reyniès A Assié G Rickman DS Tissier F Groussin L René-Corail F Dousset B Bertagna X Clauser E Bertherat J. Gene expression profiling reveals a new classification of adrenocortical tumors and identifies molecular predictors of malignancy and survival. Journal of Clinical Oncology 2009 27 1108–1115. (10.1200/JCO.2008.18.5678) 19139432

[16] Assié G Jouinot A Fassnacht M Libé R Garinet S Jacob L Hamzaoui N Neou M Sakat J de La Villéon B***et al***. Value of molecular classification for prognostic assessment of adrenocortical carcinoma. JAMA Oncology 2019 5 1440–1447. (10.1001/jamaoncol.2019.1558) 31294750 PMC6624825

[17] Mohan DR Lerario AM Else T Mukherjee B Almeida MQ Vinco M Rege J Mariani BMP Zerbini MCN Mendonca BB***et al***. Targeted assessment of G0S2 methylation identifies a rapidly recurrent, routinely fatal molecular subtype of adrenocortical carcinoma. Clinical Cancer Research 2019 25 3276–3288. (10.1158/1078-0432.CCR-18-2693) 30770352 PMC7117545

[18] Fragoso MCBV Almeida MQ Mazzuco TL Mariani BMP Brito LP Gonçalves TC Alencar GA Lima Lde O Faria AM Bourdeau I***et al***. Combined expression of BUB1B, DLGAP5, and PINK1 as predictors of poor outcome in adrenocortical tumors: validation in a Brazilian cohort of adult and pediatric patients. European Journal of Endocrinology 2012 166 61–67. (10.1530/EJE-11-0806) 22048964

[19] Turnbull AK Selli C Martinez-Perez C Fernando A Renshaw L Keys J Figueroa JD He X Tanioka M Munro AF***et al***. Unlocking the transcriptomic potential of formalin-fixed paraffin embedded clinical tissues: comparison of gene expression profiling approaches. BMC Bioinformatics 2020 21 30. (10.1186/s12859-020-3365-5) PMC6988223 31992186

[20] Stell A Sinnott R. The ENSAT registry: a digital repository supporting adrenal cancer research. Studies in Health Technology and Informatics 2012 178 207–212. (10.3233/978-1-61499-078-9-207) 22797043

[21] Dobin A Gingeras TR. Optimizing RNA-Seq mapping with STAR. Methods in Molecular Biology 2016 1415 245–262. (10.1007/978-1-4939-3572-7_13) 27115637

[22] Love MI Huber W Anders S. Moderated estimation of fold change and dispersion for RNA-seq data with DESeq2. Genome Biology 2014 15 550. (10.1186/s13059-014-0550-8) PMC4302049 25516281

[23] Gaujoux R Seoighe C. A flexible R package for nonnegative matrix factorization. BMC Bioinformatics 2010 11 367. (10.1186/1471-2105-11-367) PMC2912887 20598126

[24] Hänzelmann S Castelo R Guinney J. GSVA: gene set variation analysis for microarray and RNA-seq data. BMC Bioinformatics 2013 14 7. (10.1186/1471-2105-14-7) PMC3618321 23323831

[25] Subramanian A Tamayo P Mootha VK Mukherjee S Ebert BL Gillette MA Paulovich A Pomeroy SL Golub TR Lander ES***et al***. Gene set enrichment analysis: a knowledge-based approach for interpreting genome-wide expression profiles. PNAS 2005 102 15545–15550. (10.1073/pnas.0506580102) 16199517 PMC1239896

[26] Lê S Josse J Husson F. FactoMineR: an R package for multivariate analysis. Journal of Statistical Software 2008 25 1–18. (10.18637/jss.v025.i01)

[27] Friedman J Hastie T Tibshirani R. Regularization paths for generalized linear models via coordinate descent. Journal of Statistical Software 2010 33 1–22. (10.18637/jss.v033.i01) 20808728 PMC2929880

[28] Duregon E Volante M Cappia S Cuccurullo A Bisceglia M Wong DD Spagnolo DV Szpak-Ulczok S Bollito E Daffara F***et al***. Oncocytic adrenocortical tumors: diagnostic algorithm and mitochondrial DNA profile in 27 cases. American Journal of Surgical Pathology 2011 35 1882–1893. (10.1097/PAS.0b013e31822da401) 21989346

[29] Wong DD Spagnolo DV Bisceglia M Havlat M McCallum D Platten MA. Oncocytic adrenocortical neoplasms – a clinicopathologic study of 13 new cases emphasizing the importance of their recognition. Human Pathology 2011 42 489–499. (10.1016/j.humpath.2010.08.010) 21237489

[30] Tissier F Aubert S Leteurtre E Al Ghuzlan A Patey M Decaussin M Doucet L Gobet F Hoang C Mazerolles C***et al***. Adrenocortical tumors: improving the practice of the Weiss system through virtual microscopy: a National Program of the French Network INCa-COMETE. American Journal of Surgical Pathology 2012 36 1194–1201. (10.1097/PAS.0b013e31825a6308) 22790860

[31] Papathomas TG Pucci E Giordano TJ Lu H Duregon E Volante M Papotti M Lloyd RV Tischler AS van Nederveen FH***et al***. An international Ki67 reproducibility study in adrenal cortical carcinoma. American Journal of Surgical Pathology 2016 40 569–576. (10.1097/PAS.0000000000000574) 26685085

[32] Gara SK Lack J Zhang L Harris E Cam M Kebebew E. Metastatic adrenocortical carcinoma displays higher mutation rate and tumor heterogeneity than primary tumors. Nature Communications 2018 9 4172. (10.1038/s41467-018-06366-z) PMC6178360 30301885

[33] Jouinot A Lippert J Fassnacht M de La Villeon B Septier A Neou M Perlemoine K Appenzeller S Sibony M Gaujoux S***et al***. Intratumor heterogeneity of prognostic DNA-based molecular markers in adrenocortical carcinoma. Endocrine Connections 2020 9 705–714. (10.1530/EC-20-0228) 32698135 PMC7424337

[34] Lippert J Appenzeller S Liang R Sbiera S Kircher S Altieri B Nanda I Weigand I Gehrig A Steinhauer S***et al***. Targeted molecular analysis in adrenocortical carcinomas: a strategy toward improved personalized prognostication. Journal of Clinical Endocrinology and Metabolism 2018 103 4511–4523. (10.1210/jc.2018-01348) 30113656

[35] Liang R Weigand I Lippert J Kircher S Altieri B Steinhauer S Hantel C Rost S Rosenwald A Kroiss M***et al***. Targeted gene expression profile reveals CDK4 as therapeutic target for selected patients with adrenocortical carcinoma. Frontiers in Endocrinology 2020 11 219. (10.3389/fendo.2020.00219) PMC7176906 32373071

[36] Plaska SW Liu CJ Lim JS Rege J Bick NR Lerario AM Hammer GD Giordano TJ Else T Tomlins SA***et al***. Targeted RNAseq of formalin-fixed paraffin-embedded tissue to differentiate among benign and malignant adrenal cortical tumors. Hormone and Metabolic Research 2020 52 607–613. (10.1055/a-1212-8803) 32791542 PMC7880170

[37] Mehine M Khamaiseh S Ahvenainen T Heikkinen T Äyräväinen A Pakarinen P Härkki P Pasanen A Bützow R Vahteristo P. 3’RNA sequencing accurately classifies formalin-fixed paraffin-embedded uterine leiomyomas. Cancers 2020 12 E3839. (10.3390/cancers12123839) PMC7766537 33352722

[38] Kim HD Park S Jeong S Lee YJ Lee H Kim CG Kim KH Hong SM Lee JY Kim S 4-1BB delineates distinct activation status of exhausted tumor-infiltrating CD8+ T cells in hepatocellular carcinoma. Hepatology 2020 71 955–971. (10.1002/hep.30881) 31353502 PMC7154753

[39] Ferronika P Hof J Kats-Ugurlu G Sijmons RH Terpstra MM de Lange K Leliveld-Kors A Westers H Kok K. Comprehensive profiling of primary and metastatic ccRCC reveals a high homology of the metastases to a subregion of the primary tumour. Cancers 2019 11 E812. (10.3390/cancers11060812) PMC6628027 31212796

