# Supplementary material for: Transcriptome in paraffin samples for the diagnosis and prognosis of adrenocortical carcinoma
Source: Eur J Endocrinol. 2022 Mar 10;186(6):607–17. doi: 10.1530/EJE-21-1228 (PMC9066577; doi:10.1530/EJE-21-1228)

Supplementary Data

Supplementary Table S1. Individual patient clinical data.

See Excel File SupplTableS1.xlsx

Supplementary Table S2. Individual transcriptome data.

Raw read counts are provided for all detectable transcripts.

See csv File SupplTableS2.csv

Supplementary Table S3. Gene signatures for calculating adrenal differentiation score, proliferation score and immune score.

Adrenal differentiation score	Proliferative score			Immune score			
ABCB1	ASPM	ECT2	NUF2	ADAP1	EPOR	JAK1	PRKCE
AOX1	AURKA	EZH2	OIP5	AKAP1	ETS1	JAK3	PTPN11
ATP4A	AURKB	FAM54A	PIF1	ANGPT1	FASLG	KDR	PTPN6
CYP11A1	BIRC5	FEN1	PLK1	ARAP3	FCER1A	KITLG	PTPRC
CYP11B1	BLM	GMPS	PPIL5	ARRB2	FCER1G	LAT2	PTPRJ
CYP11B2	BUB1	GPSM2	PRC1	B2M	FCGR2B	LCK	PTPRO
CYP17A1	BUB1B	GTPBP4	PSMD14	BMX	FGR	LCP2	PXN
CYP21A2	BYSL	GTSE1	PSMD2	BTK	FOXO3	LYN	RAB7A
DLK1	C13orf34	H2AFX	RAD51	CCND3	GAB1	MAP2K4	RASA1
FDX1	C15orf23	HDGF	RECQL4	CCR5	GNG2	MAPK3	RICTOR
FDXR	C15orf42	HMGA1	RRM2	CD247	GRAP2	MITF	SDCBP
HOXA5	C1orf112	HN1	SAC3D1	CD28	GRB10	MLST8	SGK1
HSD3B2	CCNB2	IQGAP3	SNRPA1	CD3D	GRB14	MS4A2	SH2B3
INHA	CDC20	KIAA0101	SNRPD1	CD3E	GRB7	MYOF	SH3BP2
MC2R	CDC25A	KIF11	SNRPG	CD3G	GZMA	NOS2	SLA2
MGARP	CDC7	KIF14	SPAG5	CD4	HCK	NOS3	SMPD1
NOV	CDCA3	KIF15	STRA13	CD86	HCLS1	PAG1	STAP1
NR5A1	CDCA5	KIF23	TACC3	CD8A	HLA-A	PIK3C2B	STAT3
SCARB1	CDT1	KIF2C	THOC4	CD8B	HLA-DRA	PIK3CB	STAT4
SLC16A9	CENPA	KIFC1	TMEM97	CDH5	HLA-DRB1	PIK3CD	STAT5A
SOAT1	CENPF	KPNA2	TPX2	CDKN1A	HSP90AB1	PIK3CG	STAT5B
STAR	CENPM	LMNB1	TRIP13	CISH	IKBKB	PIK3R1	STAT6
SULT2A1	CEP55	LMNB2	TROAP	CSF2RB	IL12RB1	PIK3R5	SYK
TBX3	CHEK1	MCM10	TYMS	CTNNA1	IL18RAP	PLCB2	TBX21
TM7SF2	CKS1B	MCM6	UBE2C	CXCL12	IL2RA	PLCB3	TEK
	CNIH4	MKI67	UBE2S	DAPP1	IL2RB	PRF1	TNF
	DBF4	MYBL2	UBE2T	DOK1	IL2RG	PRKAA1	VAV1
	DDX39	NCAPH	UBE2V2	DOK2	ITGA3	PRKAA2	VPS4A
	DNAJC9	NEK2	UCK2	DUSP1	ITGA4	PRKCA	WAS
	DONSON	NOL11	ZWINT	ELF2	ITGA5	PRKCB	ZAP70
	DTYMK			EOMES	ITK		

Supplementary Table S4. Differentially expressed genes between transcriptome groups.

See csv File SupplTableS4.csv

Supplementary Table S5. Gene set enrichment analyses between transcriptome groups using HALLMARK annotations.

See Excel File SupplTableS5.xlsx

Supplementary Table S6. Differentially expressed genes between oncocytic and classic ACT.

See Excel File SupplTableS6.xlsx

Supplementary Table S7. Association between molecular class and clinical variables in ACC patients.

	C1A	C1B	p-value
**Age (y)**	48 (20-80)	48 (24-81)	0.47
**Sex**			1
Female	43 (80%)	24 (63%)	
Male	11 (20%)	14 (37%)	
**Cortisol secretion**			0.001
No	12 (27%)	23 (64%)	
Yes	33 (73%)	13 (36%)	
**ENSAT stage**			0.07
I-II	29 (54%)	29 (76%)	
III	15 (28%)	7 (18%)	
IV	10 (19%)	2 (5%)	
**Ki-67 proliferation index**			$<10^{-7}$
< 10%	9 (18%)	22 (63%)	
10-19 %	7 (14%)	10 (29%)	
≥ 20%	34 (68%)	3 (9%)	
**Resection status**			0.0002
R0	36 (67%)	37 (97%)	
R1-R2-RX	18 (33%)	1 (3%)	

Supplementary Figure S1. Survival according to FFPE transcriptome classification in the training cohort

A. Disease-free survival according to the predicted transcriptome class in stage I-III ACC patients

B. Overall survival according to the predicted transcriptome class in stage I-IV ACC patients

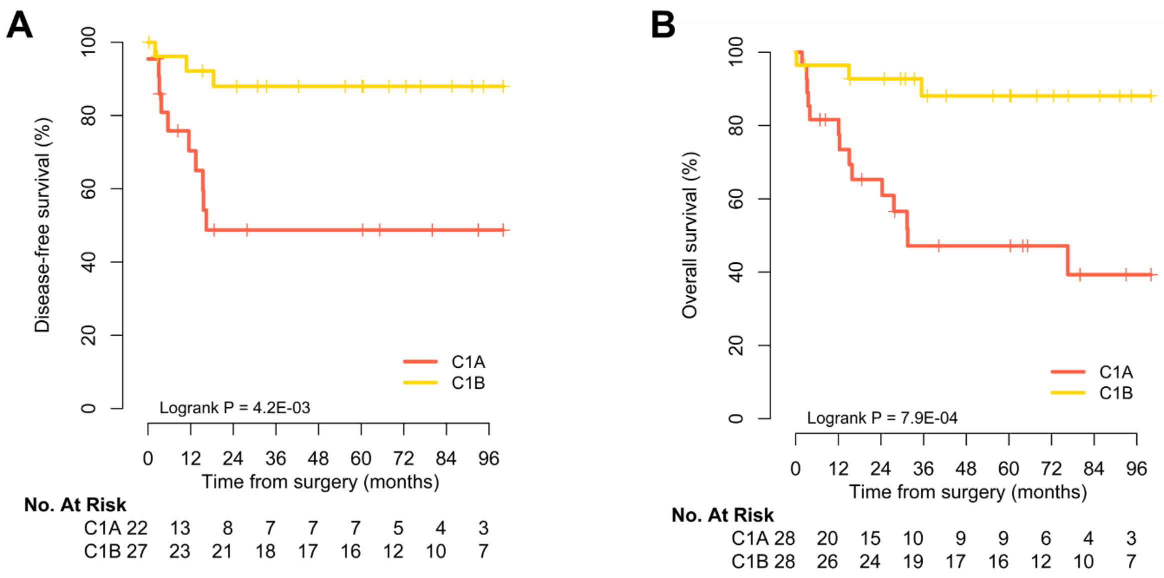

Supplement: Supplementary Data [file supplementary_material.pdf]
